# A UK national study of prevalence and correlates of adopting or not adopting a recovery identity among individuals who have overcome a drug or alcohol problem

**DOI:** 10.1186/s13011-023-00579-2

**Published:** 2023-11-17

**Authors:** Ed Day, Ifigeneia Manitsa, Amanda Farley, John F. Kelly

**Affiliations:** 1 Institute for Mental Health, School of Psychology, University of Birmingham, Edgbaston, B152TT UK; 2 Institute for Applied Health Research, University of Birmingham, Edgbaston, B152TT UK; 3 Recovery Research Institute, Center for Addiction Medicine, Department of Psychiatry, Massachusetts General Hospital, Boston, MA USA

**Keywords:** Alcohol use disorder, Drug use disorder, Recovery, Problem resolution, Identity, Adult, General population

## Abstract

**Background:**

The concept of recovery has increasingly become an organizing paradigm in the addiction field in the past 20 years, but definitions of the term vary amongst interested groups (e.g. researchers, clinicians, policy makers or people with lived experience). Although professional groups have started to form a consensus, people with lived experience of alcohol or drug (AOD) problems use the term in a different way, leading to confusion in policy making in the UK. Greater knowledge about the prevalence and correlates of adopting a recovery identity amongst those who have overcome an AOD problem would inform clinical, public health, and policy communication efforts.

**Methods:**

We conducted a cross-sectional nationally representative survey of individuals resolving a significant AOD problem (n = 1,373). Weighted analyses estimated prevalence and tested correlates of label adoption. Qualitative analyses summarized reasons for adopting or not adopting a recovery identity.

**Results:**

The proportion of individuals currently identifying as being in recovery was 52.4%, never in recovery 28.6%, and no longer in recovery 19.0%. Predictors of identifying as being in recovery included current abstinence from AOD, formal treatment, recovery support service or mutual-help participation, and history of being diagnosed with AOD or other psychiatric disorders. Qualitative analyses found themes around not adopting a recovery identity related to low AOD problem severity, viewing the problem as resolved, or having little difficulty in stopping.

**Conclusions:**

Despite increasing use of the recovery label and concept in clinical and policy contexts, many resolving AOD problems do not identify in this manner. These are most likely to be individuals with less significant histories of impairment secondary to AOD and who have not engaged with formal or informal treatment systems. The understanding of the term recovery in this UK population did not completely align with abstinence from alcohol or drugs.

## Background

The term ‘recovery’ is defined and used in different ways by people with lived experience of alcohol or drug (AOD) problems [1, 2], researchers [3, 4], clinicians and policy makers [5, 6]. Kelly and Hoeppner have proposed a conceptual basis for the recovery construct based on a bi-axial formulation [7]. The key substance-related component (‘‘remission’’) is placed on one axis, and the positive consequences ensuing from, as well as supporting, the achievement of remission (‘recovery capital’) are placed on the other axis. As remission from substance use increases, so does the extent of available recovery resources. These two axes now form the basis for most definitions of recovery, but with different levels of emphasis placed on each axis depending on the perspective taken.

When considering the first axis, definitions from the late twentieth century aligned closely with the 12-Step fellowships (e.g. Alcoholics Anonymous, Narcotics Anonymous), where recovery is associated with not just remission but lifelong complete abstinence from all substances. However, recent follow-up studies of people receiving treatment for alcohol use disorder have shown that those that return to moderate alcohol use do as well on measures of biopsychosocial functioning as those that remain abstinent [8]. Clinicians have noted that abstinence may be perceived as a high bar that discourages people from seeking treatment for AOD problems [5]. Therefore, recent recovery definitions formulated by researchers, clinicians and policy makers tend to refer to voluntary *control* of problem substance use and/or remission from diagnostic symptoms of alcohol or drug use disorder rather than only abstinence [4, 6, 8].

People with personal experience of overcoming AOD problems have tended to focus more on the second axis. For example, as part of the process to develop a Patient Reported Outcome Measure (PROM) for recovery from AOD problems, Neale and colleagues conducted focus groups with ex-users exploring 76 potential measures of recovery developed by clinicians and academics [2]. Recovery was considered a highly individualized experience, and a process rather than a state. Its definition depended on the type of addiction, the stage of recovery, and gender and other personal circumstances. Although recovery required effort and could not be measured by easy gains, the experience of achieving it was motivating, interesting and positive in some circumstances. The second axis therefore involves achievements in a range of life areas, including interpersonal relationships, housing, health, employment, self-care, use of time, community participation and well-being [5, 6]. This includes both symptom remission and building of ‘recovery capital’ [7], the ‘resources and capacities that enable growth and human flourishing’ [9].

This bi-axial conceptualisation of recovery is useful and has helped to scaffold a growing consensus on the meaning of recovery amongst researchers and clinicians [5, 6]. In the past 20 years recovery has become a core principle within the professional AOD treatment sector, resulting in a move towards ‘recovery-oriented’ services [10]. However, the understanding of the term by policy makers and the general public has been less clear, and there is evidence that the concept has been used in different ways in different geographical regions. For example, McKeganey’s analysis of policy in the USA and UK in the period between 2008 and 2014 shows that the term recovery evolved in different ways in the two countries [11]. In the USA recovery has been seen as positive addition to professional treatment services. The term has been developed and promulgated by the recovery community itself, promoted by writers such as William White [12, 13]. In contrast, in England and Scotland the media and political pressure groups defined it in contrast to the perceived predominance of harm reduction-based services. With the austerity measures introduced after the international financial crisis in 2008/9, the term recovery came to be synonymous with abstinence, which was perceived as the main goal of people attending treatment services [14].

Not all individuals who resolve significant AOD problems adopt or maintain an identity as a person in recovery [15]. A nationally representative survey of the US population reported that only 45% of individuals who had overcome an AOD problem currently identified as being in recovery, with 39.5% saying that they had never been in recovery. A further 15.4% had once been in recovery but no longer were [15]. Here we use a nationally representative sample of individuals who report overcoming an alcohol or drug problem to estimate the prevalence of different recovery identities in the UK. Following the methodology of Kelly et al. [15] we examine differences in demographic characteristics, clinical profiles, treatment and recovery support service use histories, and current wellbeing and functioning, among three distinct groups of individuals resolving a significant AOD problem who identify as (a) in recovery; (b) not in recovery; and (c) no longer in recovery. In addition, we review, categorize, and discuss qualitative descriptions of the self-reported reasons why individuals do not self-identify as being in recovery, or once did but no longer identify in this way.

## Methods

### Sampling and data collection methods

#### Eligibility

The UK National Recovery Survey was modelled on a similar process conducted in the USA in 2017 [16, 17]. The target population was the general population in the United Kingdom (England, Scotland, Wales and Northern Ireland) aged 18 or over who perceived that they had overcome a problem with drugs or alcohol. The survey was conducted by the market research and data analytics company YouGov, and ethical approval was obtained from the University of Birmingham Science, Technology, Engineering and Mathematics Ethical Review Committee (ERN_21_0565).

#### Recruitment

In stage 1 the screening question ‘Did you use to have a problem with drugs or alcohol, but no longer do?’ was administered on a UK nationally representative telephone omnibus survey in December 2021. The question was run twice to generate 2,000 responses. This provided (a) an estimate of the prevalence of AOD problem resolution and (b) the demographic profile (such as age, gender, social grade, region) of everyone who reported problem resolution. These data were used to create representative sample frames of those who have resolved a problem with AOD, which were then used to sample and weight the data.

Stage 2 involved the administration of the screening question on YouGov’s online panel of 400,000 active panellists in the UK in January 2022, allowing them to send the survey to those who qualified. YouGov employ an active sampling method, drawing a sub-sample from its panel that is representative of the group in question in terms of socio-demographics. YouGov has a proprietary, automated sampling system that invites respondents based on their profile information and how that aligns with targets for surveys that are currently active. Respondents are automatically, randomly selected based on survey availability and how that matches their profile information. Respondents are contacted by email and invited to take part in an online survey without knowing the subject at this stage. A brief, generic email invitation was used which informed the respondent only that they were invited to a survey. This helped to minimise bias from those opting in/out based on level of interest in the survey topic. Following this, the full survey was administered online, and the final sample consisted of n = 1,373 UK adults. All participants gave informed consent via the YouGov webpage prior to completing the survey.

#### Weighting

Weighting adjusted the contribution of individual respondents to aggregated figures and is used to make surveyed populations more representative of a larger, project-relevant population by forcing it to mimic the distribution of that larger population’s significant characteristics. The weighting tasks happened at the tail end of the data processing phase on cleaned data. YouGov used RIM (Random Iterative Method) weighting as its standard approach, as there were a number of different standard weights that all needed to be applied together. This weighting method calculated weights for each individual respondent from the target and achieved sample sizes for all of the quota variables. RIM weighting is an iterative process, whereby the weights are recalculated several times until the required degree of accuracy is reached. The samples were weighted to be representative of all UK adults who had overcome an AOD problem by age, gender, region and social grade (ABC1 C2DE [18]) based on the initial nationally representative telephone survey in stage 1.

### Measures

#### Recovery identity

Participants were grouped on their responses to questions about being ‘in recovery’. Firstly, they were asked “Do you consider yourself to be in recovery?” and given the option of responding ‘yes’ or ‘no’. Participants answering ‘yes’ were categorized as “currently in recovery.” Participants answering “no” were asked the follow-up question “Did you ever consider yourself to be in recovery?”, also with a yes/no response option. Participants responding “yes” were categorized as ‘used to be in recovery.’ Participants responding “no” were categorized as ‘never in recovery.’ No definition of recovery was given in the survey, and so the definition used in each case was self-determined.

#### Qualitative questions about being in recovery

Participants who indicated having never been ‘in recovery’ were asked, “You indicated that you once had a problem with alcohol or drugs but you no longer do, and you have never considered yourself to be ‘in recovery’. What is the main reason why you have never considered yourself to be ‘in recovery’?” Participants who indicated no longer being in recovery were asked: “You indicated that you once considered yourself to be in recovery but no longer do. Why is that?” Both were given an unlimited word count text box in which to type their response.

#### Alcohol or drug use and recovery-related characteristics

Participants responded to items from the Form-90 [19] about a list of substances (alcohol, cannabis, cocaine, heroin, other opioids, amphetamines, benzodiazepines, hallucinogens, synthetic drugs, and ‘others’). They were asked 1) whether they considered each substance had ever been a problem, 2) age of first use (from which we dichotomized as < 15 vs. ≥15 years) and 3) to select a primary problem substance [20]. Participants were also asked how long it had been since resolving their problem (split into three groups: 0–5 years; 5–15 years; 15 + years). The survey included items about history of 18 psychiatric disorders including alcohol use disorder and other drug use disorder (“Which of the following substance use and/or mental health conditions have you ever been diagnosed with?“) [21]. Criminal justice history was assessed with an item adapted from the Form-90 [19], ‘Have you ever been arrested?’. Possible responses included ‘no’, ‘yes – in the past year’ and ‘yes – but not in the last year’.

#### Demographics

Sex, age, and ethnicity were all captured as part of the YouGov panel process.

#### Use of recovery support services or treatment services

Participants were asked “Which of the following recovery support services or treatment programs have you ever participated in?” We grouped the nine response options into (a) used formal treatment (i.e., primary care, specialist outpatient addiction treatment, inpatient alcohol/drug detoxification services or residential rehabilitation), and (b) used recovery support services (i.e., sober living environment, recovery school, university recovery programs/communities, faith-based recovery services such as those provided by a church, synagogue, or mosque, or local peer-led recovery organization (LERO)). Participants were also asked “Which of the following self-help groups have you ever attended to help you with your alcohol or drug problem?.” We coded endorsement of any such group (e.g., AA, SMART Recovery, ‘other’) as ‘used mutual help group’.

#### Indices of psychological well-being and functioning

Quality of life was assessed using the EUROHIS-QOL [22], a widely used eight-item measure of quality of life adapted from the World Health Organization measure on quality of life. Items are rated on a 5-point Likert scale ranging from 1 (*very dissatisfied*) to 5 (*very satisfied*), with larger values indicating greater quality of life. In addition, we assessed happiness and self-esteem using single-item, 5-point Likert measures, with larger values indicating greater happiness/self-esteem, respectively [23, 24], and psychological distress using the Kessler-6 [25], a six-item scale where participants rate how often they experienced mental health difficulties (e.g., nervousness and depression) during the previous 30 days on a 5-point Likert scale ranging from 0 (*none of the time*) to 4 (*all of the time*).

#### Recovery capital

The 10-item Brief Assessment of Recovery Capital (B-ARC) [26] is a brief version of the Assessment of Recovery Capital (ARC) scale [27]. Participants reported level of agreement (1 = strongly disagree to 6 = strongly agree) with statements on their recovery, environmental support, and well-being (e.g., “I regard my life as challenging and fulfilling without the need for using drugs or alcohol”). The total score is between 10 and 60, with higher scores representing more overall Recovery Capital. This measure has demonstrated excellent concurrent validity with the longer recovery capital measure (r = .92) as well as excellent internal consistency (a = 0.95) [26].

### Statistical analysis

We calculated weighted frequencies and cross-tabulations by recovery identity group to provide a descriptive comparison of participants who consider themselves to be in recovery versus not. To identify factors associated with identifying as being ‘in recovery’ we compared the three recovery status groups (i.e., 1 - currently in recovery, 2 - used to be in recovery, and 3 – never in recovery) in univariate multinomial regression models. The univariate predictor variables included demographic, substance use, mental health, criminal justice, recovery support system use variables, quality of life and recovery capital indices. Analyses were exploratory and we did not control for multiple testing. To provide an indication of the strength of association between each tested univariate predictor and identifying as being in one of the three recovery status groups we calculated pseudo *r*-squared values of the overall model, where larger values represent stronger associations. In addition, we also provided odds ratios and 95% confidence intervals (CI) for each pairwise comparison of the three groups (i.e., currently vs. never, used to be vs. never, and currently vs. used to be). All analyses were conducted using SPSS version 29.

To provide insight into why participants self-identified in the way they did regarding recovery status, we coded the responses to the open-ended recovery questions. Two authors (ED and IM) reviewed the open-ended responses and applied the coding structure created by Kelly et al. for summarizing responses [15]. Discrepancies between coders (5.0% for “no longer;” 4.8% for “never”) were resolved by consensus in a meeting between the two coders. Results were summarized by computing weighted frequencies.

## Results

### Prevalence of recovery identity

Weighted prevalence estimates indicated 52.4% of study participants were currently ‘in recovery’; 28.6% reported never being in recovery, and 19.0% were previously in recovery but no longer were. Therefore 71.4% had been in recovery at some point, but over a quarter of this group (26.6%) dropped the recovery label with time. Of the participants reporting being currently in recovery, 47.2% were abstinent, 7.5% were abstinent from their primary problem substance only, and 45.5% were using their primary and other substances. In contrast, of the participants who had never considered themselves to be in recovery, 27.9% were abstinent, 9.5% were abstinent from their primary problem substance only, and 62.7% were using their primary and other substances. The responses from the group that used to be in recovery looked similar to the group that had never been in recovery as 27.2% were abstinent, 6.7% were abstinent from their primary problem substance only, and 66.1% were using substances. Two thirds (66.0%) of all the participants that were abstinent were currently in recovery, 13.2% used to be on recovery, and 20.9% had never been in recovery.

### Factors associated with self-identifying as ‘currently’ versus ‘no longer’ versus ‘never’ in recovery

When the three recovery groups (i.e., currently in recovery, used to be in recovery, never in recovery; Table 1) were compared, five of the demographic, substance use or clinical factors emerged as the strongest correlates of the 3-group multinomial dependent variable as determined by pseudo r^2^ value; having used recovery support services (r^2^ = 0.08), having been diagnosed with a substance use disorder (r^2^ = 0.07), having used formal treatment (r^2^ = 0.05), having attended a mutual-help group (r^2^ = 0.05), and being abstinent from all substances (r^2^ = 0.05). The strongest association was with the measure of recovery capital (r^2^ = 0.12), but none of the quality of life indices were significantly related to being ‘currently’ versus ‘used to be’ versus ‘never’ in recovery.

**Table 1:** Comparison of individuals self-labelling as ‘being in recovery’ versus “used to be” versus “never” in recovery

	Currently in recovery (n = 693; 52.4%)		Used to be in recovery (n = 251; 19.0%)		Never in recovery (n = 379; 28.6%)			
**Variable**	**%**	***SE***	**%**	***SE***	**%**	***SE***	***p***	***r*** ^***2***^
**Demographics**								
Gender							0.00	0.01
Male	62.5	2.07	74.9	3.06	64.7	2.86		
Female	37.5	2.07	25.1	3.06	35.3	2.86		
**Age**							< 0.001	0.02
18–24 (emerging adulthood)	7.5	1.36	10.3	2.45	13.0	2.43		
25–49 (young adults)	59.1	2.13	65.5	3.44	65.4	2.94		
50–64 (mid-life stage adults)	21.9	1.64	17.9	2.52	14.8	1.81		
65+ (older adults)	11.6	1.21	6.3	1.54	6.9	1.41		
**Ethnicity**							0.05	0.01
White	89.4	1.44	89.4	2.45	93.6	1.40		
Ethnic Minority	10.6	1.44	10.6	2.45	6.4	1.40		
**Substance use**								
**Time since problem resolution (in years)**							0.59	0.00
0–5 years	53.1	2.27	50.0	3.90	55.0	3.49		
> 5–15 years	31.8	2.11	35.7	3.77	32.9	3.46		
> 15 years	15.1	1.45	14.3	2.34	12.1	1.85		
**Number of substances ever identified as a problem**							0.03	0.01
1 substance	64.8	2.14	58.1	3.86	67.1	3.10		
2 substances	18.0	1.68	23.3	3.53	20.9	2.72		
3 + substances	17.2	1.72	18.6	2.99	12.0	2.19		
**Age of onset of first substance**							0.56	0.00
< 15 years of age	54.1	2.21	57.7	3.69	53.6	3.13		
≥ 15 years of age	45.9	2.21	42.4	3.69	46.4	3.13		
**Age of onset of problem substance**							0.99	0.00
< 15 years of age	41.1	2.22	41.5	3.74	40.9	3.15		
≥ 15 years of age	58.9	2.22	58.5	3.74	59.1	3.15		
**Primary substance**							< 0.001	0.03
Alcohol	62.1	2.19	53.0	3.78	54.0	3.16		
Cannabis	16.1	1.71	26.1	3.43	23.9	2.77		
Cocaine	5.3	0.98	3.1	1.26	8.7	1.86		
Opiates (heroin, other opiates)	7.3	1.14	5.8	1.72	5.0	1.3		
Amphetamine	3.2	0.79	7.4	2.05	4.2	1.15		
Other (benzodiazepines, hallucinogens, new psychoactive substances)	6.0	1.14	4.7	1.65	4.3	1.42		
**Current substance use**							< 0.001	0.05
Abstinent from all drugs/alcohol	47.0	1.90	25.9	2.80	27.2	2.30	< 0.001	0.05
Abstinent from primary problematic substance	54.5	1.90	32.7	3.00	36.5	2.50	< 0.001	0.04
**Mental health (lifetime mental health disorder diagnoses)**								
Alcohol or other drug use disorder	31.8	2.06	19.3	2.90	9.5	1.80	< 0.001	0.07
Mood disorder	25.1	2.01	28.1	3.41	16.8	2.22	< 0.001	0.01
Anxiety disorder	39.6	2.19	39.8	3.69	27.5	2.79	< 0.001	0.02
PTSD	14.9	1.58	10.7	2.05	7.2	1.82	< 0.001	0.01
Other mental health disorder	11.9	1.34	10.8	2.22	11.9	1.78	0.89	0.00
Never been diagnosed	21.6	1.84	24.5	3.31	35.6	3.02	< 0.001	0.02
**Record of arrest**							< 0.001	0.02
Never been arrested	56.6	2.23	59.1	3.7	62.0	3.10		
Yes – not in the last year	37.7	2.18	39.4	3.67	37.0	2.18		
Yes – in the last year	5.7	1.08	1.4	0.91	1.0	0.59		
**Use of recovery support**								
Used formal treatment	36.8	2.11	33.7	3.68	15.3	2.36	< 0.001	0.05
Used recovery support services	30.7	2.06	23.2	3.32	6.23	1.53	< 0.001	0.08
Used mutual-help groups	33.0	2.04	28.5	3.49	12.4	2.26	< 0.001	0.05
**Quality of life (M, SD)**								
Recovery Capital	4.45	0.83	4.29	0.93	4.23	0.85	< 0.001	0.12
Quality of life (8 items)	25.3	6.93	24.6	7.00	25.9	7.34	0.09	0.00
Happiness (1 item, 1–5 scale)	3.12	1.01	3.0	1.13	3.08	1.15	0.53	0.00
Self-esteem (1 item, 1–5 scale)	2.60	1.25	2.6	1.20	2.60	1.22	0.61	0.00
Psychological distress (Kessler 6)	9.50	6.0	9.9	6.20	9.20	6.16	0.39	0.00

Pairwise comparisons of the three recovery groups (see Table 2) showed that being in recovery was more likely than never having been in recovery if the individual was abstinent, had ever been diagnosed with an alcohol or substance use disorder, mood or anxiety disorder, had been arrested in the past year, or had used any form of assisted recovery pathway (treatment, mutual help or recovery support service). Self-identifying as being currently in recovery was less likely if there had been less than three problem substances, if the primary problem was cannabis or cocaine compared with alcohol, or if the individual had never received a diagnosis of a mental illness. Lifetime use of formal treatment or mutual-help groups played a relatively minor role in differentiating between participants self-identifying as ‘currently’ versus ‘used to be’ in recovery, but this may largely be a function of age, and relatedly, time since problem resolution. However, lifetime use of recovery support services was associated with being ‘currently in recovery’ when compared to the ‘used to be in recovery’ group.

**Table 2:** Univariate odds ratios of the pairwise comparisons of the three groups of recovery identity

	Currently in recovery versus never in recovery	Used to be in recovery versus never in recovery	Currently in recovery versus used to be in recovery
Variable	*OR* (95% CI)	*OR* (95% CI)	*OR* (95% CI)
**Demographics**			
**Gender**			
Male	1.00 [Reference]	1.00 [Reference]	1.00 [Reference]
Female	1.11 [0.85, 1.44]	0.62 [0.43, 0.88]**	1.80 [1.30, 2.49]**
**Age**			
18–24 (emerging adulthood)	0.34 [0.19, 0.62]**	0.87 [0.40, 1.90]	0.40 [0.19, 0.81]*
25–49 (young adults)	0.54 [0.34, 0.87]*	1.09 [0.57, 2.10]	0.50 [0.28, 0.88]*
50–64 (mid-life stage adults)	0.89 [0.52, 1.52]	1.32 [0.63, 2.76]	0.67 [0.36, 1.26]
65+ (older adults)	1.00 [Reference]	1.00 [Reference]	1.00 [Reference]
**Ethnicity**			
White	1.00 [Reference]	1.00 [Reference]	1.00 [Reference]
Ethnic Minority	1.74 [1.08, 2.81]*	1.74 [1.08, 2.81]*	1.00 [0.63, 1.60]
**Substance use**			
**Time since problem resolution (in years)**			
0–5 years	0.77 [0.50, 1.16]	0.70 [0.46, 1.30]	0.99 [0.64, 1.55]
5–15 years	0.77 [0.49, 1.19]	0.92 [0.53, 1.59]	0.83 [0.52, 1.32]
15 + years	1.00 [Reference]	1.00 [Reference]	1.00 [Reference]
**Number of substances ever identified as a problem**			
1 substance	0.67 [0.46, 0.97]*	0.56 [0.35, 0.89]*	1.20 [0.81, 1.76]
2 substances	0.59 [0.38. 0.93]*	0.72 [0.42, 1.23]	0.83 [0.52, 1.31]
3 + substances	1.00 [Reference]	1.00 [Reference]	1.00 [Reference]
**Age of onset of first substance**			
< 15 years of age	1.00 [Reference]	1.00 [Reference]	1.00 [Reference]
≥ 15 years of age	0.98 [0.76, 1.26]	0.85 [0.62, 1.17]	0.87 [0.65, 1.16]
**Age of onset of problem substance**			
< 15 years of age	1.00 [Reference]	1.00 [Reference]	1.00 [Reference]
≥ 15 years of age	0.99 [0.77, 1.28]	0.99 [0.77, 1.28]	0.99 [0.74, 1.33]
**Primary substance**			
Alcohol	1.00 [Reference]	1.00 [Reference]	1.00 [Reference]
Cannabis	0.59 [0.43, 0.81]**	1.12 [0.76, 1.64]	0.53 [0.37, 0.77]**
Cocaine	0.53 [0.32, 0.87]*	0.37 [0.16, 0.82]*	1.44 [0.65, 3.20]
Opiates (heroin, other opiates)	1.28 [0.74, 2.23]	1.19 [0.58, 2.44]	1.10 [0.58, 1.99]
Amphetamine	0.67 [0.34, 1.30]	1.80 [0.89, 3.65]	0.37 [0.19, 0.71]**
Other (benzodiazepines, hallucinogens, new psychoactive substances)	1.20 [0.66, 2.18]	1.09 [0.50, 2.39]	1.10 [0.56, 2.17]
**Current substance use**			
Abstinent from all drugs/alcohol	2.37 [1.81, 3.10]**	0.94 [0.66, 1.35]	2.52 [1.83, 3.46]**
Abstinent from problematic substance only	2.10 [1.61, 2.69]**	0.84 [0.60, 1.18]	2.47 [1.82, 3.35]**
**Mental health (lifetime mental health disorder diagnoses)**			
Alcohol or substance use disorder (vs. not)	4.46 [3.04, 6.50]**	2.29 [1.44, 3.64]**	1.95 [1.37, 2.78]**
Mood disorder (vs. not)	1.66 [1.20, 2.28]**	1.94 [1.32, 2.86]**	0.85 [0.62, 1.18]
Anxiety disorder (vs. not)	1.74 [1.32, 2.28]**	1.75 [1.25, 2.45]**	1.00 [0.74, 1.34]
PTSD (vs. not)	2.25 [1.45, 3.50]**	1.54 [0.88, 2.69]	1.46 [0.93, 2.30]
Other mental health disorder (vs. not)	1.00 [0.68, 1.47]	0.90 [0.54, 1.48]	1.11 [0.70, 1.77]
Never been diagnosed (vs. any diagnosis)	0.50 [0.38, 0.66]**	0.59 [0.41, 0.84]**	0.85 [0.60, 1.19]
**Record of arrest**			
Never been arrested	1.00 [Reference]	1.00 [Reference]	1.00 [Reference]
Yes – not in the last year	1.12 [0.86, 1.45]	1.12 [0.80, 1.56]	1.00 [0.74, 1.35]
Yes – in the last year	6.16 [2.11, 18.01]**	1.48 [0.34, 6.42]	4.18 [1.38, 12.63]*
**Use of recovery support**			
Used formal treatment (vs. not)	3.63 [2.67, 4.94]**	2.82 [1.92, 4.13]**	1.03 [0.77, 1.38]
Used recovery support services (vs. not)	6.72 [4.30, 10.51]**	4.55 [2.73, 7.58]**	1.48 [1.06, 2.06]*
Used mutual-help groups (vs. not	3.16 [2.35, 4.25]**	2.81 [1.87, 4.24]**	1.21 [0.90, 1.63]
**Quality of life Indices**			
Recovery Capital	1.35 [1.16, 1.57]**	1.07 [0.90, 1.29]	1.24 [1.05, 1.46]*
Quality of life (8 items)	0.99 [0.97, 1.01]	0.98 [0.95, 1.00]*	0.99 [0.97, 1.01]
Happiness (1 item, 1–5 scale)	1.03 [0.92, 1.16]	0.96 [0.83, 1.11]	0.93 [0.82, 1.06]
Self-esteem (1 item, 1–5 scale)	0.96 [0.86, 1.06]	0.94 [0.83, 1.07]	0.99 [0.88, 1.11]
Psychological distress (Kessler 6)	1.01 [0.99, 1.03]	1.02 [0.99, 1.05]	1.01 [0.99, 1.04]

*Note.* OR = odds ratio, 95% CI = 95% confidence interval

* *p* < .05. ** *p* < .01

Recovery capital was quantified using the B-ARC, which includes 10 items that reflect substance use and sobriety; global psychological health; global physical health; citizenship; social support; meaningful activities; housing and safety; risk taking; coping and life functioning; and recovery experience [26]. Pairwise comparisons showed that participants that reported being ‘in recovery’ reported higher B-ARC scores than those who had ‘never been in recovery’ (OR 1.35, 1.16–1.57) and people who ‘used to be in recovery but no longer are’ (1.24, 1.05–1.46). There was no significant difference between those who used to be in recovery and those were never in recovery.

### Qualitative feedback on reasons for perceived recovery status

Respondents described why they considered themselves to have “never” been in recovery or to be “no longer” in recovery (Table 3). The most common reason for “never” having been in recovery was respondents’ ability to stop using substances and in most cases without the use of any external support (27.8%; e.g., *“I gave up overnight and that was the end of it.”*). This stemmed from a range of factors, including their ability to recognise their problematic use (e.g., “*I got on top of the problem myself quickly.”*) and lack of enjoyment of substance use (e.g., *“I stopped enjoying taking cocaine so stopped taking it”*). The second most common reason for “never” having been in recovery was continued substance use but not at problematic levels (25.8%; e.g., *“I still drink but moderately now.”*). Respondents mentioned that they stopped or significantly reduced their use of alcohol and/or substance (e.g., *“I think I’ve normalised my drinking now, but I no longer take drugs or smoke.”*) and were also able to control their use (e.g., *“I am now able to just have one drink and be satisfied with it.”*). Another important reason was the low severity of their self-reported alcohol and/or substance use (20.8%). Here respondents explained that their substance use never caused significant impairment (e.g., *“I don’t believe my addiction was bad enough.”*) or they stopped using before reaching that point (e.g., *“I stopped when I saw habitual problems arising. I never got to a place of true addiction.”*).

**Table 3:** Reasons for not identifying as being in recovery

Category (coding instructions)	Verbatim quotes		*%*		*SE*
**Never in recovery (** ***n*** ** = 379)**					
**Ability to stop** (e.g., participants made the decision to quit; this happened for various reasons, including no longer enjoying substance use, recognising the problem, and financial issues)	“I just got to a point where I didn’t want to continue with it so removed myself from the environment.” “I found a way to change my behaviour by acknowledging that the previous attitude was problematic.” “I just really liked it but couldn’t afford it, so I stopped.”		27.8		3.25
**Some use but not at problematic level** (e.g., can control use; alcohol and drug use have reduced)	“I still consume alcohol, but I can control it but not stop.” “Because I still drink, but highly reduced compared to what it was during the pandemic.” “Because I still use these things now and again but not to extremes and not every single day.”		25.8		3.21
**Low severity** (e.g., participants think that their substance use was never a problem; their substance use was unrelated to addiction)	“I only take drugs socially and never found it a problem.” “I wouldn’t really say my problems were severe enough to consider myself an addict and thus I wouldn’t say I’m in recovery.” “…cannabis isn’t addictive and once I had things to fill my time with such as university, I didn’t need it anymore.”		20.8		3.00
**Rejection of ‘recovery’ label** (e.g., participants never identified with the term ‘in recovery’; participants believe that the term ‘in recovery’ refers to an illness or injury that needs to be addressed rather than a behaviour)	“Never identified with that language.” “I don’t agree with the recovery rhetoric.” “Because I don’t see addiction as a disease to be treated but as a behaviour.” “I don’t like the term ‘recovery’ as if I had some injury, just changing a mindset on drinking habits.”		13.7		2.70
**Matured out** (e.g., participants refer to significant life changes such as family and health issues, as well as changes in social networks)	“I didn’t make the conscious decision to quit one day I had a disabled child and could no longer drink as had to care for them 24/7.” “Once I became pregnant none of these lifestyle choices were appropriate.” “I stopped immediately after a serious brain injury though using illegal drugs put me in a coma for 2 weeks.” “My life moved on. I changed the people I hung out with so stopped taking drugs. It wasn’t a particular decision.”		9.4		1.90
**Starting new or switching substances** (e.g., participants started using new substances and not their problem substance)	“I swapped one addiction for another.” “Just addicted to other stuff.”		3.8		1.52
**Mental health problem rather than physical addiction** (e.g., participants believe that mental health challenges caused their substance use; they relate the term ‘in recovery’ with physical dependency on drugs and alcohol)	“I was never physically addicted but rather mentally relied on it too much…” “I don’t believe I was ever an addict. The problems I had with drugs were with my mental health, my relationship with my wife and financial.”		2.4		0.89
**Unclassified/other** (e.g., provided reason is not clear or is missing)	“I miss them too much.” “Addictive personality” “[no answer]”		11.0		2.35
**Used to be in recovery (*n*** **= 251)**					
**Resolved** (e.g., fully recovered; it has been a long time since participants’ recovery; participants no longer crave substances; participants consider addiction as a problem which has been solved)	“I am fully recovered. I am totally free in mind body and spirit from the addictions I once had. I know with absolute certainty that I will never use drugs again.” “I have no need for any drugs anymore. I have kept away from most temptation for over 20 years.” “I have no inclination for drugs or alcohol anymore not for a very long time.” “I have no need or want for drugs.” “I’ve overcome the problem.”	56.6		4.25	
**Relapsing** (e.g., participants have started using substances and/or drinking again; potential reasons that participants mentioned were COVID-19, personal issues, and peer pressure)	“I slipped back.” “I have returned to using substances in a problematic way.” “Since Covid my alcohol intake has increased again.” “Getting divorced and changes in my life.” “I started hanging around with old friends again who still have drink and drug issues.”	14.2		3.45	
**Non-problematic continued use** (e.g., participants have formed a healthy relationship with alcohol and substances; they may use substances occasionally; participants can control use)	“I believe I’ve moved past it, and now have a healthy relationship with alcohol (e.g., drinking very occasionally, being able to stop at one drink).” “Because I have stopped taking drugs like I used to. Only once every 6 months now if that” “I have found a level of alcohol consumption which is non-destructive and controlled.”	13.8		2.93	
**Matured out** (significant life changes such as family circumstances; participants explained that their substance use was caused due to personal problems and difficulties that no longer exist)	“Found my happiness and have a baby boy.” “It was a long time ago and I do not have the problems and difficulties I had then.” “Because I do not have the same issues that caused me to have the addiction.”	8.2		2.39	
**Rejection of ‘recovery’ label** (e.g., participants referred to the negativity of the term ‘recovery’; difficulty in understanding the term ‘recovery’; some new terms emerged)	“I feel that the ‘addict’ label and being ‘in recovery’ are somewhat counterproductive past a certain point. I had a problem, and I overcame it. I prefer not to have my entire life dominated by that.” “It isn’t clear what this means. I have recovered and that’s that.” “I prefer smart recovery.”	2.0		1.04	
**Ability to stop without using external support** (e.g., self-treatment without using any type of external support such as medication and/or their GPs support)	“I achieved my goal myself.” “Abstained using own will power.”	1.7		0.88	
**Starting new or switching substances** (e.g., using new substances rather than their previous problematic substance)	“I have recovered from heroin addiction and no longer take it, but I do drink far too much alcohol.” “I previously had an alcohol addiction, but I recovered and no longer do. Because of physical injuries I now have a prescription opioid addiction which I currently trying to overcome.”	1.4		0.64	
**Religion** (e.g., their religious involvement helped participants helped them overcome their addictions; on the other hand, limited religious involvement led to relapse)	“Jesus healed me of my addiction.” “I left church. Got back into heavy drinking from end of 2005 especially.”	0.8		0.80	
**Unclassified/other** (e.g., provided reason is not clear or is missing)	“It was a long time ago.” “I was wrong.” “[no answer]”	9.0		2.27	

Rejection of the ‘recovery’ label was an important reason for why respondents felt they had never been in recovery (13.7%) (e.g., *“Because it implies that I endured some kind of long-standing suffering as a result of using drugs, when that isn’t the case”*). Participants also felt that they had “matured out” of substance use (9.4%), citing major life events that became more important than substance use. These major life events included personal situations (e.g., *“As I acquire a more positive self-image and came out as a gay man, I no longer felt the need for drug use.”*), as well as changes in their family (e.g., *“Met a woman, she didn’t like it and made it clear it was that or her”*) and social environment (e.g., “… *when I started a new job, I realised that I could not do the job properly under the influence of cannabis”*). Other reasons respondents never considered themselves in recovery were that they began using new substances instead of the problem substance (3.8%; e.g., *“I will always do one or the other.”*), or that their substance use were a way of coping with other mental health problems that had been resolved at the time of the survey (2.4%; e.g., *“I abused alcohol previously owing to low self-esteem, low self-confidence and severe social anxiety.”*).

The most prevalent reason for why respondents felt they were “no longer” in recovery was that their substance use was “resolved” (56.6%). Potential explanations given by participants were that they had stopped using substances and/or alcohol (e.g., *“From very early on in my recovery, I simply knew I wouldn’t get drunk again.”*), did not want to drink or use substances (e.g., *“I no longer have a need or desire to drink.”*), or no longer experienced the worst aspects of alcohol or drug use (e.g., *“Because I no longer consider myself to have an addiction and have recovered from the worst of it.”*). Similar to the respondents who “never” considered themselves to be in recovery, other reasons for “no longer” being in recovery were “non problematic use” (13.8%), “matured out” (8.2%), “rejection of recovery label” (2.0%), “starting new substances” (1.4%) and “ability to stop without using external support” (1.7%). A small but significant group of participants no longer considered themselves to be in recovery as they had “relapsed” or returned to substance use (14.2%; e.g., *“Drinking again.”, “I slipped back.”*).

## Discussion

This is the first nationally representative study in the UK to examine the prevalence and predictors of recovery identity, and to investigate in detail the self-reported reasons why many who have resolved a significant problem with alcohol or drugs (AOD) have either never chosen to adopt such a label or have stopped identifying with it over time. More than half (52.4%) of the weighted sample considered themselves to be in recovery, whereas only 28.6% never thought of themselves as in recovery. Although 71% of the weighted sample initially considered themselves to be in recovery, over a quarter of this group (26.7%) subsequently stopped using the label. These results are similar to a study conducted in the USA, where 45% of a weighted national sample who had overcome an AOD problem considered themselves to be in recovery, and a further 15.4% had once been in recovery but no longer were [15]. Even allowing for a slightly different sampling process in the two studies [15, 17], it is perhaps surprising that a larger proportion of participants identified as being in recovery in the UK sample. The UK and the USA have very different social care, treatment and criminal justice systems, and there are considerable differences in the uptake of 12-Step fellowship groups such as AA and NA [28]. Rough calculations suggest that whereas approximately 0.5% of the total US population is a member of an AA group [29], the equivalent figure in the UK is over ten times smaller (0.03% [30]). This was reflected in the 45.1% of the US sample who reported attendance at a mutual help group [16] compared with 29.7% of the UK sample [17]. As the term ‘recovery’ is strongly associated with the 12-Step fellowships it was anticipated that the term would be used more in the USA.

It is useful to think of a spectrum of engagement with AOD use in the general population, from abstinence, through unproblematic use, to the development of medical, psychological or social problems, to dependence [31]. As described in the US Surgeon General’s report on Addiction [32] (p4-4), such a spectrum requires a ‘substance use care continuum’. Our study population included a wide portion of this spectrum, allowing examination of both assisted and unassisted pathways to overcoming AOD problems [16, 17]. People who do not use the term recovery have several reasons for rejecting it. A significant proportion of this group felt that their alcohol or drug problems were of low severity, or that they had the ability to stop with minimal trouble, hence a ‘recovery’ label was not justified. Others described overcoming their AOD problems by controlling (rather than stopping) their use or switching to another substance. Some participants rejected the label altogether due to its ‘medical’ connotations, or because they believed that alcohol or drug use was a means of controlling another underlying issue such as a mental health problem.

Personal recovery is a subjective experience, and the individual’s understanding of his/her recovery may change over time [1, 33]. A significant proportion of people had moved on from their perceived recovery status, no longer finding it useful. Many described feeling that the alcohol or drug problem had resolved and would not return, often due to other positive changes in their life such as parental responsibility. Like those who never considered themselves to be in recovery, this group were less likely to use assisted pathways to help them resolve their AOD problem. This suggests that their lives may not have been as seriously impacted as the group currently in recovery, and so a salient self-label was not required as an implicit self-preservation strategy. Another explanation is that, given the stigma of an AOD history, letting go of the label could lessen the potential for future discrimination and create a more positive self-concept [34]. By dropping the recovery label, individuals may hope to distance themselves from the negative experiences and memories associated with their past substance use. As Kelly et al. conclude, “the term recovery, while adaptive, positive, and potentially helpful for many, still comes with a great deal of societal stigma, potential discrimination, and emotional distress that may lead people to not wish to identify with this concept and self-label” [15].

The strongest correlates of adopting the recovery self-concept were variables reflecting the use of treatment services, recovery support services, or mutual-help organizations. Recovery status was also associated with a lifetime diagnosis of alcohol or drug use disorder, and both sets of correlates reflect greater involvement with AOD and/or associated impairment. The concept of recovery has been linked with the idea of empowerment and self-determination, and some research has highlighted the importance of identity change processes, through which the internalised stigma and ‘spoiled identity’ is replaced with a new, positive identity [35, 36]. Adoption of this identity may be a function of degree of negative impact of the disorder, where the cognitive integration of this identity is one of self-preservation and to maintain vigilance because more is at stake for those more severely affected if they relapse [15]. Maintaining the salience of this identity is often paramount for this group, and it may also be that adopting a recovery identity is important to symbolise a new direction and new priorities in life. Social identity theory [37] describes how affiliation with significant others who share similar properties helps to develop an individual’s social perception. Social categorization into an ingroup (‘in recovery’) and an outgroup enables the world to appear ordered and simplified, allowing individuals to navigate with clearly defined rules for behaviour through their daily lives [34]. Best and colleagues have integrated these two theories into the Social Identity Model of Recovery (SIMOR), which proposes that “recovery is best understood as a personal journey of socially negotiated identity transition that occurs through changes in social networks and related meaningful activities” [36]. This twin approach of connecting with new positive networks of support through shared purposeful activity may be essential in those with the most severe forms of addiction. Self-identifying as being in recovery was also associated with increased recovery capital scores, but not markers of quality of life. This might suggest that the measure of recovery capital (B-ARC) is capturing a sense of what the individual means by defining themselves as being ‘in recovery’, and that this is more than just a general sense of quality of life or happiness.

Being abstinent from alcohol or drugs was strongly associated with being in recovery, but the picture was complicated. Not everyone who saw themselves as being in recovery was abstinent, even from their primary substance. Likewise, not everyone who was abstinent reported being in recovery. Therefore, a simple assumption that being in recovery involves being abstinent is not helpful or accurate. As the polarised dispute between harm reduction and abstinence in UK policy circles in the early 2010s shows [11, 38, 39], the reality is much more complicated. Many people find the term recovery to be helpful in forging a new identity post AOD problems, but it does not necessarily mean that they are abstinent. Likewise, building recovery capital is a worthwhile aim even if your goal is to reduce but not stop AOD use. These findings have implications for the way we communicate and label clinical and public health outreach and intervention efforts in addressing AOD problems. By giving participants the choice of either being in recovery or not we have simplified a complicated situation, where the subjective experience of change is different from external, measurable behaviours [36].

Some important limitations of this study should be considered. Self-reported resolution of an AOD problem is not synonymous with remission of an alcohol or other drug use *disorder*, although is likely to significantly overlap with it. Use of a lifetime diagnostic instrument to explore the presence/absence and severity of alcohol or drug use disorder could have reduced the subjectivity in this self-assessment. However, it is important to understand how such issues are resolved in the whole population, as less than a quarter of people who may benefit will access treatment services in their lifetime [40]. Furthermore, the bulk of the burden of alcohol or drug problems in a population is carried by those who do not meet diagnostic criteria [41]. This study is cross-sectional and correlational and so caution should be taken when making inferences about causal connections among variables over time. However, they provide a useful basis for developing future longitudinal studies.

## Conclusions

Alcohol and other drug problems create a significant medical, psychological and social burden for society in the UK. The concept of recovery is increasingly used as an organizing paradigm for treatment services, despite considerable disagreement about the use of the word in policy circles in the past decade. This nationally representative study offers insight into the prevalence and correlates of choosing to adopt or not adopt such an identity in a general population who have overcome AOD problems. Many individuals who report resolving a significant AOD problem do not identify as being ‘in recovery’, but those with the most significant problems are more likely to do so. Recovery is associated with abstinence, but many individuals who have controlled rather than stopped their AOD use also see themselves as in recovery. Individuals who do not use recovery as a self-label are less likely to engage with treatment services, and so may require novel strategies to reach, and subsequently help sustain, their positive gains over time. Witkiewitz and colleagues have described a trend towards removal of the term abstinence from professional definitions of recovery [8], and it may be that a focus on measuring changes in recovery capital over time is a better way of aligning with the lived experience of individuals who have overcome an AOD problem [42].

## Data Availability

The data that support the findings of this study are available on request from the corresponding author [ED].

## Abbreviations

AOD: Alcohol or drug
ARC: Addiction Recovery Capital (scale)
B: ARC–Brief Assessment of Recovery Capital (scale)
CI − 95%: Confidence interval
M: Mean
OR: Odds ratio
PROM: Patient Reported Outcome Measure
RIM: Random Iterative Method
SD: Standard deviation
UK: United Kingdom
USA: United States of America
